# Supplementary material for: Aberrantly Expressed MicroRNAs in Cancer-Associated Fibroblasts and Their Target Oncogenic Signatures in Hepatocellular Carcinoma
Source: Int J Mol Sci. 2023 Feb 21;24(5):4272. doi: 10.3390/ijms24054272 (PMC10002073; doi:10.3390/ijms24054272)

Supporting Information

Table S1. The binding probability of miRs to *TGFBR1* mRNA were predicted by TargetScan 8.0

miRNAs	Position in the 3'-UTR	Seed match	Context++ score	Predicted relative KD
hsa-miR-101-3p	460-466	7mer-m8	-0.29	-3.849
	3993-4000	8mer	-0.24	-3.714
hsa-miR-490-3p	3933-3939	7mer-m8	-0.17	-3.435

Table S2. Primer sequences.

Gene		Sequence
hsa-miR-101-3p		5'-TACAGTACTGTGATAACTGAA-3'
hsa-miR-490-3p		5'-CAACCTGGAGGACTCCATGCTG -3'
TGFBR1	Forward	5'-GGGGATGGGGGAAATACGAC-3'
	Reverse	5'-CCAGAGCAGCCTTCAGTCAA-3'
HMBS	Forward	5'- GGAGGGCAGAAGGAAGAAAACAG -3'
	Reverse	5'-CACTGTCCGTCTGTATGCGAG-3'
GAPDH	Forward	5'-AGTATGACAACAGCCTCAAG-3'
	Reverse	5'-TCATGAGTCCTTCCACGATA-3'

Figure S1. The qRT-PCR results of two miRs

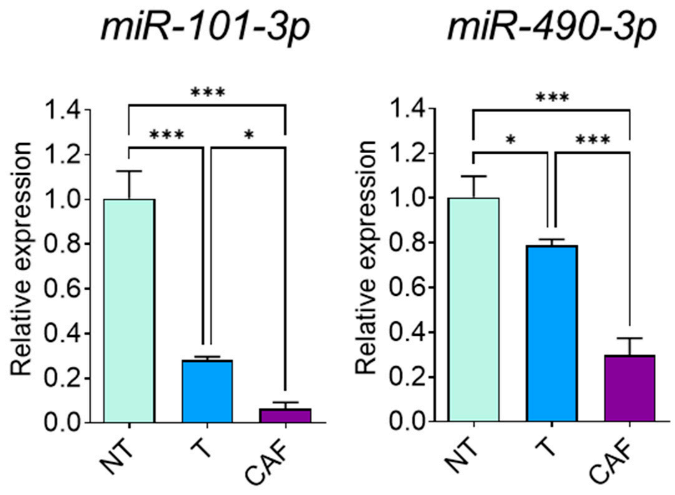

Figure S2. Confirmation of mycoplasma-free cultures in this study

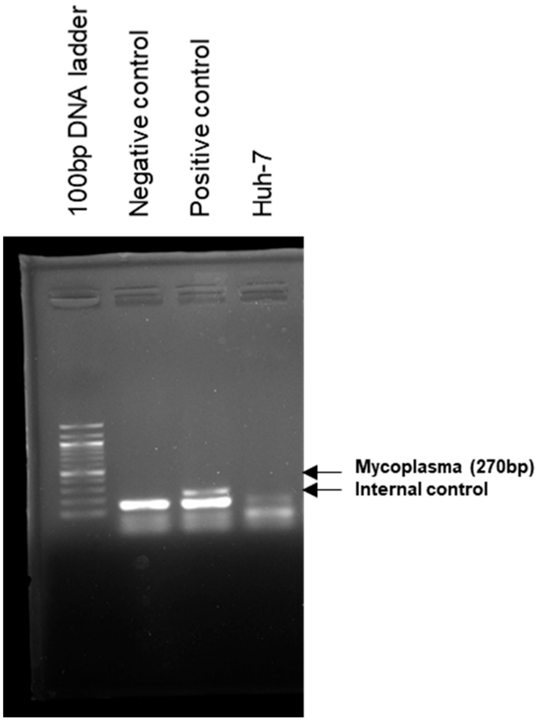

Supplement: Supplementary file 1 [file ijms-24-04272-s001.zip › ijms-2162262-supplementary.pdf]
